# Unfavorable Outcome of Neuroblastoma in Patients With 2p Gain

**DOI:** 10.3389/fonc.2019.01018

**Published:** 2019-10-09

**Authors:** Katarzyna Szewczyk, Aleksandra Wieczorek, Wojciech Młynarski, Szymon Janczar, Mariola Woszczyk, Zuzanna Gamrot, Radosław Chaber, Mariusz Wysocki, Monika Pogorzała, Mirosław Bik-Multanowski, Walentyna Balwierz

**Affiliations:** ^1^Department of Medical Genetics, Faculty of Medicine, Jagiellonian University Medical College, Krakow, Poland; ^2^Department of Pediatrics Oncology and Hematology, Faculty of Medicine, Jagiellonian University Medical College, Krakow, Poland; ^3^Department of Pediatric Oncology and Hematology, University Children's Hospital of Krakow, Krakow, Poland; ^4^Department of Pediatrics, Oncology, Hematology and Diabetology, Medical University of Lodz, Lodz, Poland; ^5^Unit of Pediatric Oncology and Hematology, City Hospital, Chorzow, Poland; ^6^Clinic of Pediatric Oncology and Hematology, Faculty of Medicine, University of Rzeszow, Rzeszow, Poland; ^7^Department of Pediatric Hematology and Oncology, Collegium Medicum, Nicolaus Copernicus University, Bydgoszcz, Poland

**Keywords:** 2p gain, MYCN gain, MYCN amplification, neuroblastoma, structural chromosomal aberrations

## Abstract

**Background:** Amplification of the *MYCN* oncogene is the most unfavorable genetic factor in neuroblastoma patients. However, knowledge about the clinical impact of low-level multiplication of *MYCN* is still insufficient. Therefore, we aimed to investigate the disease course in patients with different copy number status of *MYCN*.

**Materials and Methods:** We examined 105 children diagnosed with neuroblastoma from 2010 to 2018 in five pediatric oncology centers in Poland. We determined the *MYCN* status at diagnosis by the interphase FISH examination and assessed the clinical outcome in patients.

**Results:** A total of 35% of tumors presented with chromosome 2 numerical changes, 20% had *MYCN* amplification and 16% revealed 2p gain. Unexpectedly, we observed very low overall survival and event free survival (EFS) rates in neuroblastomas with 2p gain, which were comparable with patients with *MYCN* amplification.

**Conclusions:** The 2p gain alteration should be reported as a strong unfavorable prognostic marker in neuroblastoma patients.

## Introduction

Neuroblastoma (NB) is the most common (6–8%) solid extracranial tumor of childhood that is responsible for 50% of all childhood cancer-related deaths. The unfavorable prognosis with high risk of relapse and death in NB correlates with age over 18 months at diagnosis, advanced disease stage, and established genetic markers. Amplification of the *MYCN* oncogene (MNA) is the most robust genetic factor correlated with poor clinical outcome and can be found in about 16–20% of NB cases (and up to 40% in high-risk tumors) (1–5). Gain of the *MYCN* gene *locus* on the short arm of chromosome 2 (2p24) can also be found in NB cells (6–8). Since the oncogene *MYCN* is involved in all facets of metastasis: cell adhesion, motility, invasion, and degradation of extracellular matrix, it is crucial to accurately estimate the *MYCN* status at NB diagnosis (4, 9). The recommended technique for counting *MYCN* copies is fluorescence *in situ* hybridization (FISH) (2, 10). The international guidelines for FISH analysis clearly stratify between high-level copy number change of *MYCN* (MNA), low-level multiplication of (*MYCN* gain and 2p gain) and numerical changes and no multiplication of *MYCN* (normal status) (1, 2).

Contrary to MNA, little is known about the importance of low-level *MYCN* variants (including 2p gain) as prognostic factors in NB patients (6, 10–12). Therefore, the aim of this study was to assess the clinical impact of such variants in relation to normal *MYCN* status and MNA and to evaluate the potential, practical benefits of routine testing for low-level *MYCN* variants in all patients with NB. Additionally, for the first time we present clinical and tumor biological features in a population of Polish NB patients.

## Materials and Methods

We enrolled all patients diagnosed with NB between 2010 and 2018 year in five regional pediatric oncology centers in Poland. Patients were treated according to the current protocols of European International Society of Pediatric Oncology Neuroblastoma Group. Clinical features assessed included the age at diagnosis, disease stage, as well as the presence of metastases and relapses. We collected data about biological profiles of NB tumors, including the status of *MYCN* and *ALK locus* (*ALK* is located in close vicinity of *MYCN* and co-amplification of these two genes was observed) (13). Additionally, we assessed the presence of 11q23 deletion, which was reported to have unfavorable prognostic importance in NB and to be negatively correlated with MNA (4, 6, 14, 15). This study was carried out in accordance with the recommendations of The Ethics Committee of Jagiellonian University Medical College, Krakow, Poland. All subjects gave written informed consent in accordance with the Declaration of Helsinki.

We determined the *MYCN* status at diagnosis by means of interphase FISH examination according to international guidelines (1, 16, 17). The dual-color set of fluorescence probes N-MYC (MYCN) Amplification (Cytocell, Cambridge, UK) was hybridized to tumor imprint slides according to manufacturer's protocol. The NB patients were classified as having tumors with:

- MNA (>4-fold increase of *MYCN* signals in comparison with the reference probe),
- 2p gain (1–2 more of *MYCN* signals in comparison with the reference probe),
- chromosome 2 numerical changes (equal number of *MYCN* and reference signals corresponding to numerical alterations of chromosome 2),
- normal *MYCN* status (wild-type, two *MYCN* copies and two reference signals).

We used the interphase FISH method for detection of 11q23 deletion [KMT2A(11q23)/SE11 probe; Leica Biosystems, Wetzlar, DE] and, additionally, if archival tumor imprint slides were available, the *ALK* gene status was assessed (ALK Breakapart probe; Cytocell, Cambridge, UK).

Finally, we compared the disease outcome in specific subgroups of patients with different copy number status of *MYCN* gene. Descriptive statistics, the Fisher's exact test and Kaplan–Meier curves were implemented, and Cox's proportional hazards regression model was used for multivariate analysis.

## Results

**Figure 1:**
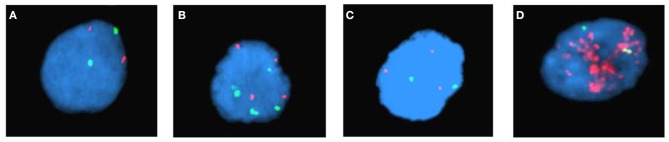
Examples of copy number status of *MYCN* gene in NB tumors detected by interphase FISH examination (probes: red-*MYCN locus-*2p24.3 and green-control *locus*-2q11.2): **(A)** normal, **(B)** chromosome 2 numerical changes, **(C)** 2p gain, **(D)** amplification.

We enrolled 105 NB patients. In 37 (35%) cases chromosome 2 numerical changes were observed in tumors, whereas in 21 children (20%) *MYCN* amplification and in 17 (16%) 2p gain were found. A group of 30 (29%) patients showed normal *MYCN* status (Figure 1).

A total of 82 NB patients completed the 5-years clinical observation period or died prior to the 5 year time point since diagnosis. The biological markers of interest and the clinical observational data for this group of children are listed in Table 1.

**Table 1:** Clinical data for the study group.

**Feature**		**Subgroup (estimation based on** ***N*** **=** **82 cases)**			
		***MYCN* normal (*N* = 21)**	**Numerical changes (*N* = 26)**	**2p gain (*N* = 17)**	MNA^*^ (*N* = 18)
Patient age	>18 months	14 (67%)	12 (46%)	10 (59%)	12 (67%)
	<18 months	7 (33%)	14 (54%)	7 (41%)	6 (33%)
	INSS^**^ 1	5 (24%)	6 (23%)	3 (18%)	2 (11%)
	INSS 2	5 (24%)	2 (8%)	0 (0%)	0 (0%)
Disease stage	INSS 3	6 (29%)	10 (38%)	3 (18%)	3 (17%)
	INSS 4	3 (14%)	6 (23%)	8 (47%)	12 (67%)
	INSS 4S	2 (9%)	2 (8%)	3 (18%)	1 (6%)
	High	4 (19%)	5 (19%)	7 (41%)	16 (89%)
Risk group	Intermediate	6 (29%)	15 (58%)	5 (29%)	2 (11%)
	Low	11 (52%)	6 (23%)	5 (29%)	0 (0%)
	Metastases	5 (24%)	8 (31%)	11 (65%)	13 (72%)
Patient outcome	Relapse	8 (38%)	7 (27%)	5 (28%)	5 (29%)
	Death	2 (9.5%)	2 (8%)	6 (35%)	5 (28%)

* MNA, MYCN amplification;

** *INSS, International Neuroblastoma Staging System*.

We found an unexpectedly high percentage of cases with NB stage 4 in children with 2p gain. This was significantly higher than in patients with *MYCN* normal status (*p* = 0.03 in Fisher's exact test). We observed that the incidence of patients with stage 4S was about two times higher in the group of 2p gain than in other subgroups, but the differences were not statistically significant. We also found a statistical trend regarding the higher percentage of deaths in patients with 2p gain (*p* = 0.06). Furthermore, no statistically significant differences were found regarding the relapse rate.

We observed a slightly higher frequency of 11q23 deletion in patients with 2p gain in comparison with other groups (Table 2). However, the differences also did not reach statistical significance. Finally, archival tumor imprint slides for *ALK* gene status assessment were available in a subset of 10 patients with 2p gain and 11 patients with MNA. *ALK* co-gain was observed in six cases with 2p gain (60%) and *ALK* co-amplification was detected in one case of MNA.

**Table 2:** The status of 11q23 deletion in the study group.

	**Subgroup (estimation based on** ***N*** **=** **53 cases)**			
**Feature**	***MYCN* normal (*N* = 12)**	**Numerical changes (*N* = 18)**	**2p gain (*N* = 10)**	MNA^*^ (*N* = 13)
11q23 deletion	1 (8%)	2 (11%)	3 (30%)	2 (15%)

* *MNA, MYCN amplification*.

**Figure 2:**
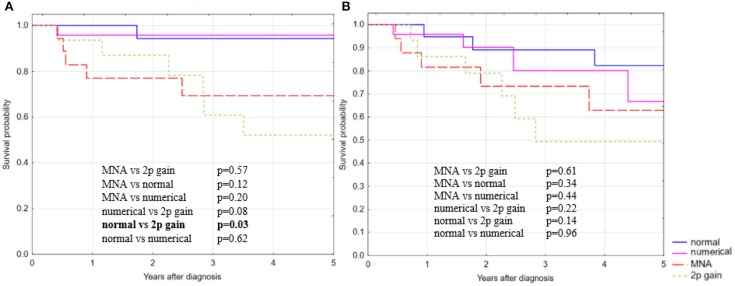
Kaplan–Meier estimates of **(A)** 5-OS with statistically significant differences, and **(B)** 5-EFS without statistically significant differences between specific subgroups in relation to copy number status of *MYCN* gene.

**Figure 3:**
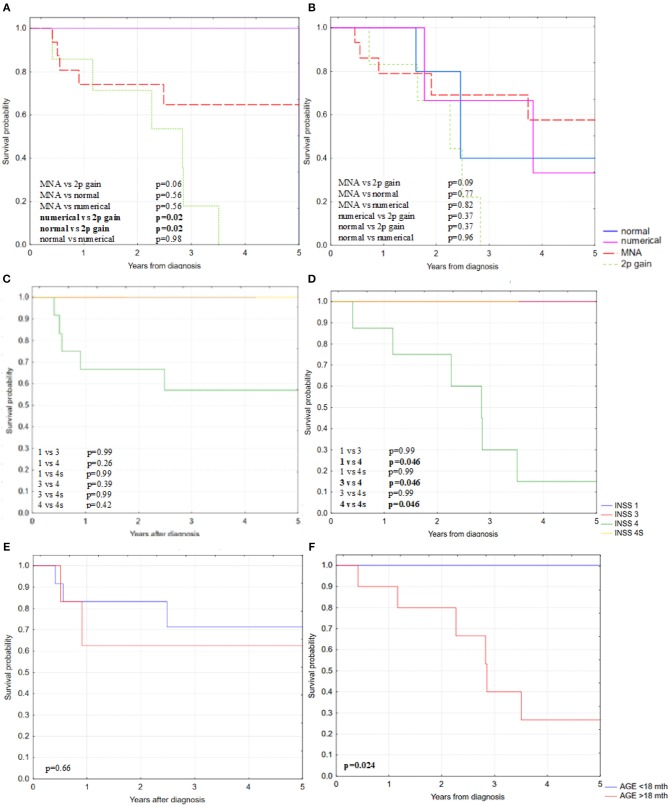
Kaplan–Meier estimates of **(A)** 5-OS and with statistically significant differences in high-risk disease group of NB patients and **(B)** 5-EFS without statistically significant differences between specific subgroups in relation to copy number status of *MYCN* gene in high-risk disease group of NB patients; **(C)** 5-OS in MNA subgroup and **(D)** 5-OS in 2p gain subgroup in relation to disease stage; **(E)** 5-OS in MNA subgroup and **(F)** 5-OS in 2p gain subgroup in relation to patients age.

The 5-year overall survival rate (OS) for patients with MNA and 2p gain reached 69 and 52%, respectively, whereas in patients with numerical changes of chromosome 2 it was similar to normal *MYCN* status (95 and 94%, respectively, Figure 2). In our cohort, patients with 2p gain in NB tumors had very low OS that was comparable to the MNA group and significantly lower than in patients with normal *MYCN* status (*p* = 0.03; Kaplan–Meier statistics). We observed a similar trend for the 5-year event free survival (EFS), but the differences did not reach statistical significance (Figure 2). 2p gain decreased survival rate specifically among patients with high-risk disease, disease stage 4 and aged over 18 months (Figure 3). The group of children with chromosome 2 numerical changes did not differ significantly from those with normal *MYCN* status with regard to disease outcome.

Although none of the statistical tests attempted for Cox's proportional hazard regression analysis reached statistical significance, we would like to summarize our approach in order to indicate where important conclusions could be reached with an increase in sample size. *MYCN* status was compared for 5-year OS and EFS (normal status as a reference). MNA, 2p gain, and chromosome 2 numerical changes gave hazard ratios (HR) of 2.46 (0.27–22.51, 95% CI, *p* = 0.43), 3.76 (0.44–32.50 95% CI, *p* = 0.23, and 1.49 (0.13–16.63, 95% CI, *p* = 0.75) for 5-year OS. Comparing NB disease stages 1, 2, 3, 4, and 4s (in accordance with INSS—International Neuroblastoma Staging System), was also carried out as was assessing the impact of age. We also tested for interaction between *MYCN* status and INSS, *MYCN* status and age, and all three together, but again none of the interacting factors reached statistical significance.

## Discussion

The incidence of 2p gain aberration that we observed in our cohort was relatively high (16%) in comparison to other European populations [3% as reported by Campbell et al. (18), 6% as submitted Cohn et al. and Spitz et al. (11, 12) and 13–14% as announced Jeison et al. and Stallings et al. (6, 7)], but comparable to Asian populations [~15% as described by Souzaki et al. (10)]. These differences may be related to the number of cases in each study group or the biological origin of the analyzed samples [some researchers evaluated the *MYCN* gene status in tumor specimens and bone marrow aspirates, without distinguishing between primary and metastatic sample origin (6, 12, 18)].

Our results show that the presence of 2p gain in patients with NB is frequently co-incident with advanced disease stage at diagnosis, confirming other previous observations (6, 10, 11). However, in contrast to MNA, NB tumors with 2p gain do not present with *locus*-specific, highly increased expression of *MYCN* (12). This indicates the existence of different mechanisms present in both groups of patients. It may be that 2p gain leads to a partial trisomy of the short arm of chromosome 2, which contains not only the *MYCN* oncogene but also several other genes involved in carcinogenesis (*ADAM17, ALK, BCL11A, DDX1, EML4, EPCAM, EPAS1, E2F6, FANCL, GALM, GREB1, ID2, MSH2, MSH6, NCOA1, NOTO, REL, RHOB, ROCK2, RRM2, SDC1, TPO, XPO1*)^1^,^2^. Multiplication of the above-listed genes in addition to the *MYCN* oncogene could be responsible for worsening of the clinical prognosis in patients with NB.

Previous reports suggested that *ALK* copy number multiplication, if occurring concurrently with *MYCN*, significantly reduces patients survival especially for the intermediate- and high-risk group (13, 19), and that 2p gain tumors encompassing the *ALK locus* also associate with worse outcome (20). The presence of additional *ALK* gain in our “2p gain” subgroup might partially explain worse disease outcome in some of these patients. However, the individual contribution of the gain of *ALK* and other above-mentioned genes to disease outcome should be investigated in larger cohorts of NB patients.

In our study, the 2p gain corresponded with decreased average 5-year OS and EFS, similarly to the group of patients with MNA (the 5-year OS was even slightly lower in the subgroup with 2p gain). This is in line with the results of Jeison et al. (6) and Campbell et al. (18). However, the above-mentioned very advanced disease stage at the time of diagnosis in many of our 2p gain-patients seems to explain the frequently observed unfavorable disease outcome.

Moreover, Stiglani et al. (21) reported that NB tumors with structural chromosome changes, such as the prognostically independent and unfavorable 11q23 deletion, have a greater tendency to accumulate additional genetic instability. Therefore, the potential coexistence of structural alterations like 2p gain and 11q23 deletion in NB cells should be considered (6, 7, 10–12). However, the frequency of 11q23 deletion was relatively low in our material, which makes detailed analysis difficult. On the other hand, Jeison et al. did not find significant differences in the 5-year OS between subgroups of 2p gain patients with or without coexistence of 11q deletions in tumor cells (6). In contrast, Cohn et al. postulated that 2p gain is not an independent prognostic factor (11).

In conclusion, our results indicate that the presence of 2p gain in patients with NB is an unfavorable prognostic marker, especially in patients with high-risk disease, similar to *MYCN* amplification. It seems that 2p gain correlates with decreased OS and, potentially might decrease EFS. Further studies in larger populations of patients are necessary to verify the clinical significance of 2p gain. If our results are confirmed, patients with 2p gain might require more intensive therapy.

## Data Availability Statement

The raw data supporting the conclusions of this manuscript will be made available by the authors, without undue reservation, to any qualified researcher.

## Ethics Statement

The studies involving human participants were reviewed and approved by The Ethics Committee of Jagiellonian University Medical College, Krakow, Poland. Written informed consent to participate in this study was provided by the participants' legal guardian/next of kin.

## Author Contributions

KS: original idea for the study. KS, AW, and MB-M: data analysis and interpretation. KS and MB-M: statistical analyses and drafting the manuscript. KS, AW, WM, SJ, MWo, ZG, RC, MP, and MWy: data acquisition. AW, WM, MWo, RC, MWy, and WB: critically revising the manuscript for important intellectual content.

### Conflict of Interest

The authors declare that the research was conducted in the absence of any commercial or financial relationships that could be construed as a potential conflict of interest.

## Footnotes

^1^http://www.cancerindex.org

^2^http://atlasgeneticsoncology.org
